# Toward standardized methods in canine vaginal microbiome research: evaluation of storage, host DNA depletion, and database selection

**DOI:** 10.1128/spectrum.00583-25

**Published:** 2025-07-08

**Authors:** Lotte Spanoghe, Guillaume Domain, Florin Posastiuc, Amanda Hettiarachchi, Adelaide Panattoni, Sebastiaan Theuns, Filip Van Immerseel, Geert Opsomer, Ann Van Soom, Penelope Banchi

**Affiliations:** 1 Department of Internal Medicine, Reproduction and Population Medicine, Faculty of Veterinary Medicine, Ghent University 573392 , Merelbeke, Belgium; 2 Department of Clinical Sciences II, Faculty of Veterinary Medicine, University of Agronomic Sciences and Veterinary Medicine 591712, Bucharest, Romania; 3 PathoSense BV, Lier, Belgium; 4 Department of Translational Physiology, Infectiology and Public Health, Faculty of Veterinary Medicine, Ghent University 366759 , Merelbeke, Belgium; 5 Laboratory of Applied Microbiology and Biotechnology, Department of Bioscience Engineering, University of Antwerp 692679, Antwerp, Belgium; 6 Department of Pathobiology, Pharmacology and Zoological Medicine, Faculty of Veterinary Medicine, Ghent University 703523, Merelbeke, Belgium; Cleveland Clinic Lerner Research Institute, Cleveland, Ohio, USA

**Keywords:** canine vaginal microbiome, microbiome, veterinary microbiology, DNA sequencing, canine reproduction

## Abstract

Standardized methods for investigating the canine vaginal microbiome are not available yet. Data using next-generation sequencing (NGS) are still limited, and methodologies lack consistency. In theory, microbiome results can be significantly affected by factors like sampling technique, storage, DNA extraction methods, and reference databases, which can all introduce bias. To address these concerns, we compared two storage methods for the sample (no medium vs. medium), examined the effect of host DNA depletion, and tested two reference databases (Emu vs. SILVA) using samples from six bitches, totaling 26 samples. Host depletion showed no significant impact on bacterial composition, nor did the storage conditions. However, when comparing reference databases, we found significant differences in beta diversity, emphasizing the importance of database choice when comparing studies. The proposed protocol paves the way for future studies on the canine vaginal microbiome, setting the basis for more precise and comprehensive microbiome profiling in this field.

**IMPORTANCE:**

Understanding the vaginal microbiome in dogs could lead to new insights into reproductive health and fertility, but progress is limited by the lack of clear guidelines on how samples should be collected, stored, and analyzed. This study helps clarify which steps in the process truly matter and which have little impact, offering practical guidance for researchers entering this field. By highlighting where inconsistencies can influence outcomes and which methodological choices affect results, we take an important step toward more reliable and comparable research. These findings support future scientific studies and hold potential to improve veterinary care over time.

## INTRODUCTION

The microbiome in the reproductive tract has been extensively investigated in women, particularly regarding its influence on fertility and reproductive health (1–3). More recently, similar investigations have been started in dogs, though this field is still in its early stages (4–8). While numerous studies have explored the canine vaginal microbiome using traditional culture techniques (9–11), only three have utilized next-generation sequencing (NGS) (4–6). This technique has revolutionized microbiome research in dogs, offering comprehensive data and a more accurate representation of bacterial diversity.

However, the advanced sensitivity of these techniques comes with challenges, as numerous steps in the process - from sampling to sequencing - may pose a risk for contamination or bias. In human microbiome research, technical studies have led to the establishment of standard operating procedures for studying the vaginal microbiome (12–14). In dogs, such research is still emerging, with the few existing studies employing diverse methodologies, from sampling techniques to sequencing approaches. For instance, cranial vaginal sampling has been performed with various tools, including cotton (5) and flocked swabs (6), with or without otoscopes (4) or speculums (6), and sometimes without any guard at all (5). Additionally, storage and processing methods are often inadequately detailed, with some studies mentioning storage at −80°C without specifying the duration (5), while others report storage at 4°C, followed by processing within 36 h (6). There are also inconsistencies in the type of receptacle or medium used for storage. Some specify microcentrifuge tubes (Eppendorf, Hamburg, Germany) (4) or fecal swab transport medium (Copan, Brescia, Italy) (6), while others omit this detail entirely (5). Notably, specialized media, such as eNAT (Copan, Brescia, Italy), are available for microbiome research, designed to stabilize nucleic acids and prevent bacterial growth by lysing cells. This medium has been shown to preserve the microbiological profile of vaginal swab samples over time and under different storage conditions in women (12).

DNA extraction methods also vary across studies, with different commercial kits in use, many of which include a host DNA depletion step. This process is designed to remove host DNA, such as that derived from epithelial cells, to increase sensitivity to bacterial DNA. However, a recent study investigating optimal methodologies for large-scale research on the microbiome of women (skin, saliva, and vagina) found that host depletion significantly reduces the abundance of gram-negative bacteria (12). Interestingly, previous studies on the canine vaginal microbiome using NGS (4, 6) did not detect sequences from the genus *Escherichia*, which has been frequently isolated using culture techniques (9–11). We hypothesized that if host DNA depletion were applied to canine vaginal samples, it could introduce a bias against gram-negative bacteria, reducing their representation in sequencing data.

Furthermore, following sequencing, the obtained reads are compared to reference databases to identify bacterial taxa. Different databases are available, each varying in size and accuracy. For example, two canine vaginal microbiome studies used the Greengenes database (5, 6, 15), while one used the SILVA database (4, 16), introducing further variability in results.

Due to the wide variation in methodologies, including storage methods, DNA extraction protocols, and sequencing techniques, it remains challenging to compare results or build cohesive knowledge on the canine vaginal microbiome. Therefore, the present study aimed to (i) compare two distinct storage methods (medium versus no medium) with regard to DNA yield and microbial composition, (ii) evaluate the impact of host DNA depletion during bacterial DNA extraction, (iii) investigate whether host DNA depletion selectively affects gram-negative bacteria, and (iv) assess how different taxonomic databases (Emu [17] and SILVA [16]) influence microbial diversity and taxonomic resolution, with the ultimate goal of establishing more standardized protocols for future research on the canine vaginal microbiome.

## MATERIALS AND METHODS

### Animals

**Fig 1:**
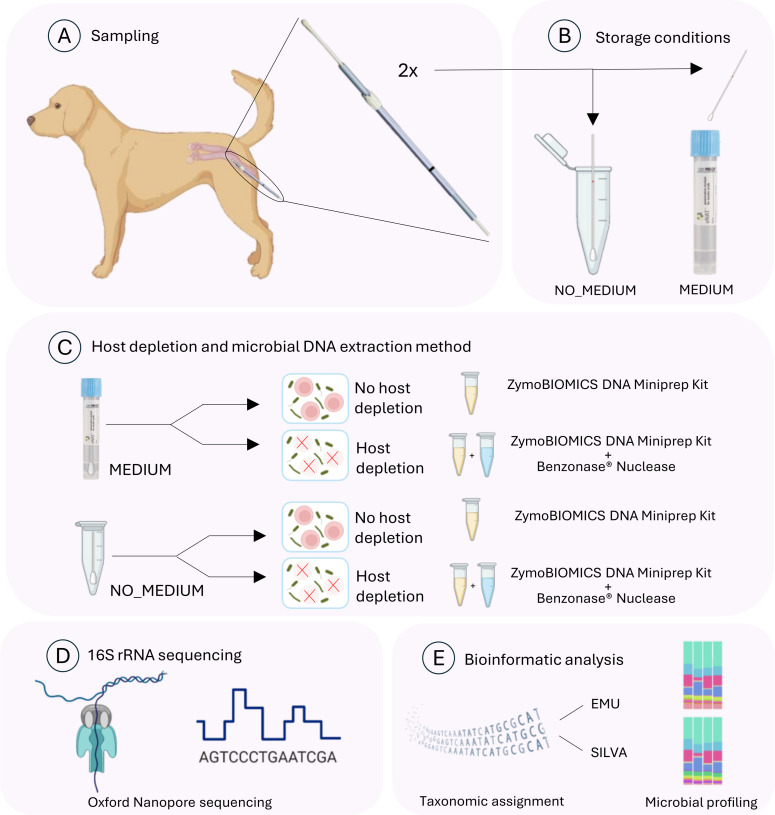
Overview of the study workflow created with BioRender.com. (**A**) Two vaginal swabs per dog were collected using sterile double guarded swabs from six bitches in estrus. (**B**) One of the swabs was stored in a sterile, PCR-clean microcentrifuge tube (Eppendorf, Hamburg, Germany), while the other one was stored in 2 mL of eNAT medium (Copan, Brescia, Italy). (**C**) For samples in the eNAT medium, two 250 µL aliquots were taken: one aliquot underwent DNA extraction with host depletion, while the second was processed without host depletion. For the samples stored in microcentrifuge tubes, 1 mL of PBS was added, and two 250 µL aliquots were extracted as described. For both protocols, the ZymoBIOMICS DNA Miniprep Kit (Zymo Research) was used. For the host-depleted samples, an additional step was included before extraction: 3.5 µL of benzonase nuclease (Merck) was added. (**D**) Full-length 16S rRNA sequencing of all samples was performed using Oxford Nanopore technology. (E) Taxonomic assignment of sequences was performed using two different databases (Emu and SILVA) with subsequent corresponding bioinformatics analysis.

Six healthy, reproductively sound bitches were presented to the Faculty of Veterinary Medicine at Ghent University for cycle follow-up and were included in the present study (Fig. 1). The bitches were sampled while in estrus and confirmed to be reproductively healthy based on medical history, clinical examination, and ultrasound of the reproductive organs (ovaries and uterus). The cohort consisted of medium-sized (15–25 kg) and large (>25 kg) breeds, including Rhodesian Ridgeback, Australian Cobberdog, Hovawart, Finnish Lapphund, White Swiss Shepherd, and Boerboel. The dogs’ age ranged from 2.5 to 7 years (mean 4.5 years; standard deviation [SD] 1.82). The cycle follow-up included blood progesterone measurement (Speed Reader, Virbac), vaginal cytology, and ovarian ultrasonography (MyLab 50 XVision System, Esaote). None of the animals had received antibiotics or immunosuppressive therapy within 6 months prior to sampling.

### Sample collection

Vaginal samples were collected using a double-guarded swab technique to prevent contamination from the vulvar lips, vestibule, and caudal vagina. Double-guarded culture swabs designed for horses (Continental Plastic Corp., Wisconsin, USA) were modified in length, sterilized, and refitted with a FLOQSwab (Copan, Brescia, Italy). Swabs from the cranial vagina were obtained using sterile gloves. Two swabs were taken from each bitch during the same consultation, timed as close to ovulation as possible based on blood progesterone values, vaginal cytology, and ultrasound of the ovaries. Non-sterile swabs for vaginal cytology were collected after the two double-guarded swabs were taken to avoid contamination. One swab was placed in a tube containing 2 mL of the eNAT medium (Copan, Brescia, Italy), while the other was stored in a sterile, PCR-clean 1.5 mL microcentrifuge tube (Eppendorf, Hamburg, Germany). For samples stored in the eNAT medium, the swab was transferred aseptically by breaking the swab at the 8 mm breakpoint. For those placed in microcentrifuge tubes, the swab was cut to the desired length using sterile scissors. The microcentrifuge tubes used were PCR-clean, individually wrapped, and sterilized prior to use to minimize the risk of contamination. To avoid sampling bias, in three randomly selected bitches, the first swab was placed in the eNAT medium, while for the remaining three, the first swab was stored in the microcentrifuge tube.

All samples, including those collected in microcentrifuge tubes and in the eNAT medium, were immediately stored at –20°C after collection and subsequently transferred to a –80°C freezer within 15 to 30 min following the conclusion of the consultation. Samples remained at –80°C until DNA extraction. These conditions are consistent with previously published protocols that have demonstrated minimal impact on microbial composition when samples are rapidly frozen and stored at –80°C (18, 19).

This study was approved by the Ethical Committee for Animal Experiments of the Faculty of Veterinary Medicine and the Faculty of Bioscience Engineering at Ghent University (approval number: 2023-089) and conducted in accordance with national and institutional guidelines for the ethical use of animals in research. Written informed consent was obtained from all dog owners prior to sample collection.

### DNA extraction

DNA extraction and 16S rRNA gene amplicon sequencing were outsourced to PathoSense BV (Lier, Belgium). All samples were stored at –80°C immediately after collection and remained frozen until DNA extraction was performed. To ensure consistency, DNA extraction was carried out for all samples at the same time after the full set had been collected. For samples in the eNAT medium, two 250 µL aliquots were taken after vortexing for 30 seconds: one aliquot underwent DNA extraction with host depletion, while the second was processed without host depletion. For the samples stored without medium, 1 mL of PBS was added and vortexed for 30 seconds, and two 250 µL aliquots were extracted as described. For DNA extraction, the ZymoBIOMICS DNA Miniprep Kit (Zymo Research) was used. For host depletion, 3.5 µL of benzonase nuclease (Merck) was added to the 250 µL aliquot and incubated at 37°C for 30 minutes. The enzymatic reaction was then halted by adding 12.5 µL of 10 nM EDTA. DNA extraction was performed on the remaining 250 µL aliquot. The DNA yields were quantified using a Quantus fluorometer (Promega), and the DNA quality was evaluated via a NanoDrop 2000 spectrophotometer (Thermo Scientific) and agarose gel electrophoresis. All samples were subjected to full-length (V1–V9) 16S rRNA gene amplification with 30 cycles prior to nanopore sequencing.

### 16S rRNA gene sequencing

Library preparation was performed using the ligation sequencing kit, SQK-LSK114 (Oxford Nanopore Technologies [ONT], UK) following the targeted full-length 16S rRNA gene amplification. Sequencing libraries were run on MinION flow cells (R10.4.1) using a GridION device (ONT, UK) for 24 hours. Raw data were collected and base-called using the Dorado basecaller (v. 7.3.11). Raw sequencing reads were demultiplexed and filtered by quality (*Q* score > 10) and length (1,300–1,700 bp). Taxonomic assignment was performed twice using the Emu bioinformatics tool (17): first with its default curated Emu database (17) and second with the SILVA SSU database (v138.1) (16). Reads assigned to bacterial taxa detected in negative control samples (no medium and medium) were subtracted from those assigned to the same bacterial taxa in the swab samples. The remaining reads were used to calculate the relative abundances of bacterial phyla, families, genera, and species.

### Bioinformatics and statistical analyses

To assess potential differences in microbial profiles between samples stored with and without medium, alpha and beta diversity analyses were performed. Initially, all samples were compared, regardless of the host depletion status. Subsequently, comparisons between storage conditions were made for both host- and non-host-depleted samples. Alpha diversity was estimated as the Shannon diversity, Chao1, Faith, and observed richness indices, with statistical significance between the medium and no medium groups determined via the Wilcoxon rank sum test. Beta diversity was evaluated using Bray-Curtis dissimilarity and Unweighted UniFrac to assess bacterial community differences between samples. A principal coordinates analysis (PCoA) plot was generated to visualize the clustering of samples. Significant differences in community dissimilarity between the two storage conditions were evaluated using permutational ANOVA (PERMANOVA) performed with 9,999 permutations using the adonis2 function from the *vegan* package in R. The same alpha and beta diversity analyses were applied to compare microbial profiles between host- and non-host-depleted samples, as well as between the reference databases used (Emu vs SILVA). Data were visualized in R using the ggplot2 (v. 3.5.1) package (20), while statistical analyses were performed with the vegan (v. 2.6.6.1) (21) and phyloseq (v. 1.46.0) packages (22).

Finally, linear discriminant analysis effect size (LEfSe) was employed to identify bacterial species with significantly different relative abundances between the groups. LEfSe analyses were conducted to compare host- and non-host-depleted samples, as well as the Emu and SILVA databases, within both the medium and no medium groups. Bacterial taxa with an LDA log score > 2.0 were considered significant. These analyses were conducted using the R package microbiomeMarker (v1.8.0) (23).

## RESULTS

### The canine vaginal environment is low in biomass, and DNA concentrations and read numbers do not differ between storage methods

A total of 12 swab samples (six stored in the eNAT medium and six in microcentrifuge tubes) were each divided into two aliquots, one subjected to host depletion and one not, resulting in 24 samples. Additionally, two negative controls (one in the eNAT medium and one in a microcentrifuge tube) were included, increasing the total number of sequenced samples to 26. All samples were analyzed using full-length 16S rRNA gene amplicon sequencing. The initial DNA yield prior to sequencing averaged 0.524 ng/µL (standard deviation [SD]: 1.033), indicating that the vaginal environment is low in biomass. The DNA yields ranged from 0.0352 to 4.49 ng/µL for the swab samples, while the negative controls yielded between 0.0057 and 0.0334 ng/µL. Following targeted PCR amplification (30 cycles), the DNA concentration significantly increased, with an average post-PCR yield of 78.208 ng/µL (SD: 32.5). Pre-PCR DNA concentrations did not differ significantly between storage conditions, as determined by the Wilcoxon signed-rank test (Table 1).

**TABLE 1:** Comparison of the pre-PCR DNA concentrations and sequencing reads between storage conditions^*a*^

Variable	Storage condition	Md	IQR	*P*-value
DNA concentration (ng/µL)	M_NoHD	0.256	0.221	0.917
	NM_NoHD	0.160	0.177	
	M_HD	0.229	0.128	0.116
	NM_HD	0.090	0.249	
Number of reads per sample	M_NoHD	151,816.5	8,262.5	0.463
	NM_NoHD	144,408.5	9,051.75	
	M_HD	171,755	21,819	0.116
	NM_HD	164,858.5	14,077	

^*a*^ The results of the Wilcoxon signed-rank test comparing pre-PCR DNA concentrations and the number of sequencing reads per sample across different storage conditions (medium [M] vs. no medium [NM]) with and without host DNA depletion (NoHD and HD) are shown. Median (Md) values, interquartile ranges (IQR), and corresponding *P*-values are presented. No statistically significant differences were observed between storage conditions for either pre-PCR DNA concentrations or sequencing reads.

**Fig 2:**
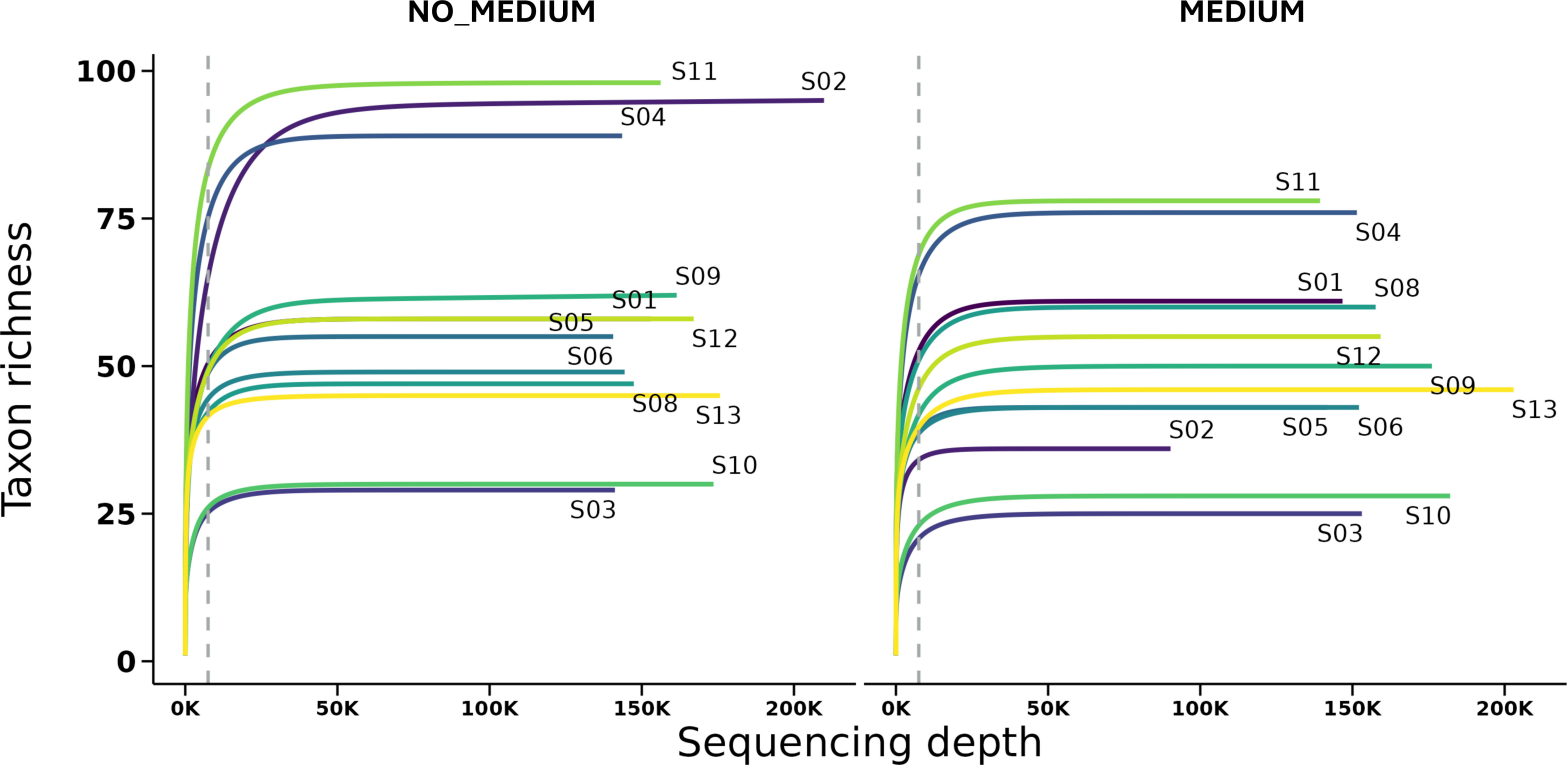
Rarefaction curves for the 16S rRNA gene sequences at the species level of all samples.

Nanopore sequencing generated a total of 3,819,638 16S rRNA reads, with an average of 159,152 (SD: 23,731) reads per sample and an N50 read length of 1,484 (SD: 2) nucleotides. The number of reads per sample did not differ significantly between storage conditions (Wilcoxon signed-rank test; Table 1). The negative controls yielded a total of 114,978 reads, with an average of 42,572 reads per control. To account for potential contamination, reads assigned to bacterial taxa detected in the negative control samples were subtracted from those in the corresponding swab samples, resulting in a final total of 3,704,660 reads for downstream analysis. As shown in Fig. 2, rarefaction curves reached a plateau across all samples, confirming sufficient sequencing depth.

### Storage method and host DNA depletion do not significantly affect microbial diversity or composition

**Fig 3:**
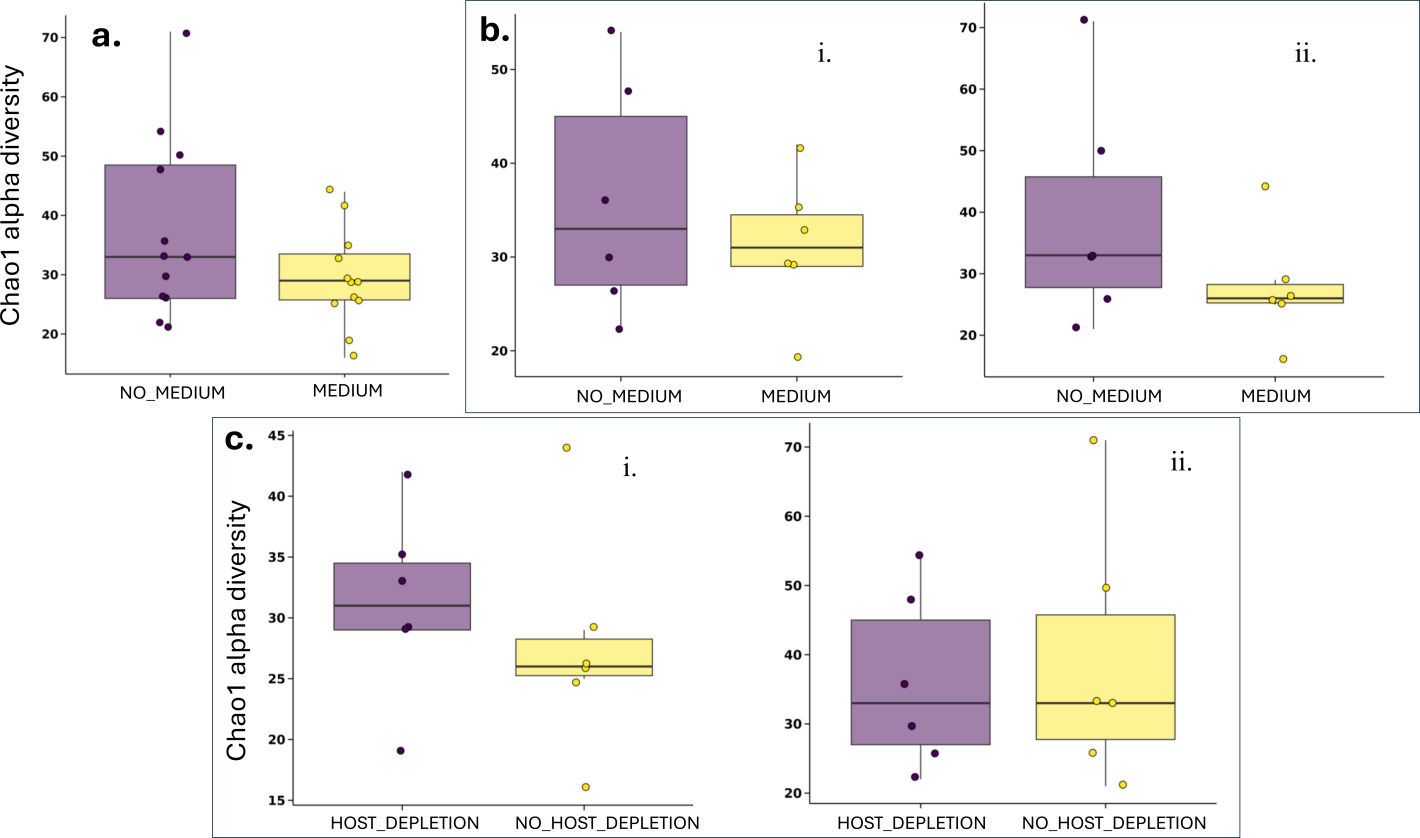
Alpha diversity is not significantly different based on storage conditions and host depletion. Box plots of alpha diversity (Chao1-index) at the genus level comparing storage and processing conditions. **a**. Comparison of storage conditions (medium vs. no medium) using all samples. **b**. Comparison of storage conditions (medium vs. no medium) for host-depleted (i) and non-host-depleted samples (ii). **c**. Comparison of processing conditions (host depletion vs. no host depletion) for samples stored with (i) and without medium (ii). No significant differences in alpha diversity (observed richness, Chao1, Shannon index) were found (*P* > 0.05).

Alpha diversity (the variety of species within a single sample or community) assessed using observed richness, Shannon, Faith, and Chao1 (Fig. 3) indices revealed no significant differences between sample groups (Wilcoxon rank sum test, *P*-values > 0.05 for all comparisons).

**Fig 4:**
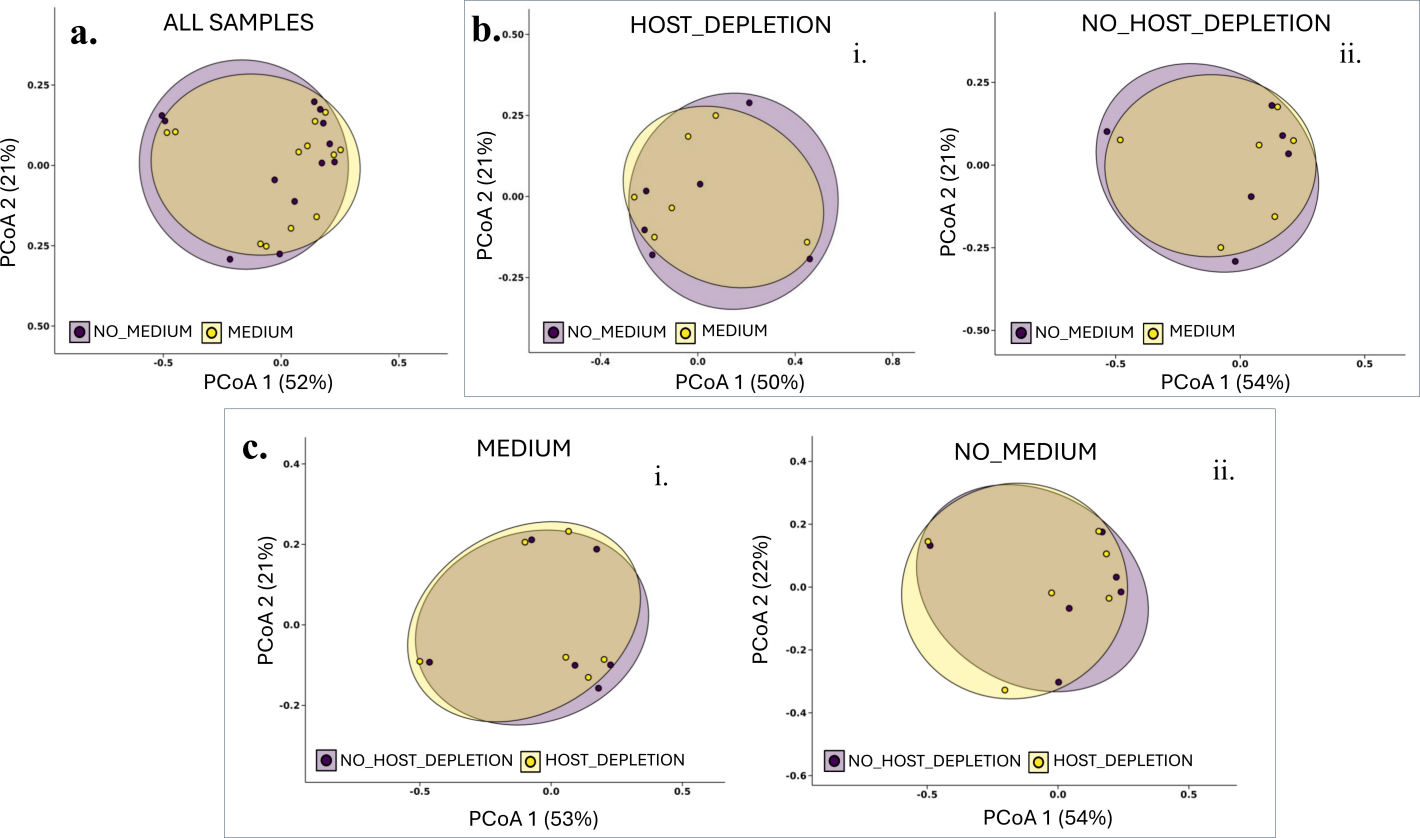
Beta diversity is not significantly different based on storage conditions and host depletion**.** The principal coordinate analysis (PCoA) plots are based on the Bray-Curtis distance-matrix at the genus level. **a**. Comparison of storage conditions (medium vs. no medium) using all samples. **b**. Comparison of storage conditions (medium vs. no medium) for host-depleted (i) and non-host-depleted samples (ii). **c**. Comparison of processing conditions (host depletion vs. no host depletion) for samples stored with (i) and without medium (ii). No significant differences in beta diversity (Bray-Curtis dissimilarity index and Unweighted Unifrac) were found using a permutational ANOVA (PERMANOVA) test with 9,999 permutations (*P* > 0.05).

Beta diversity analysis (compares the differences in species composition between samples or communities) using Bray-Curtis dissimilarity and Unweighted UniFrac showed no significant community differentiation between the two storage conditions nor the host DNA depletion status (PERMANOVA test, 9,999 permutations, *P* > 0.05). Principal Coordinates Analysis (PCoA) plots were used to visualize beta diversity (Fig. 4).

All diversity analyses were conducted using taxonomic annotations obtained through the EMU algorithm. Although both EMU- and SILVA-based annotations were evaluated, neither showed significant differences in alpha or beta diversity; for consistency, only the EMU-based results are presented in the figures.

LEfSe was used to identify the bacterial species with a significantly different relative abundance between the different groups evaluated. LEfSe analysis was performed comparing host- and non-host-depleted groups on medium and on no medium samples separately. Only significant taxa with an LDA log score > 2.0 were considered in the results. From this analysis, no significantly different bacterial species were found between the treatments.

### Reference database selection significantly affects taxonomic classification and community structure

Alpha diversity assessed using observed richness, Shannon, Faith, and Chao1 indices revealed no significant differences between the reference databases used (Wilcoxon rank sum test, *P*-value > 0.05 for all comparisons).

**Fig 5:**
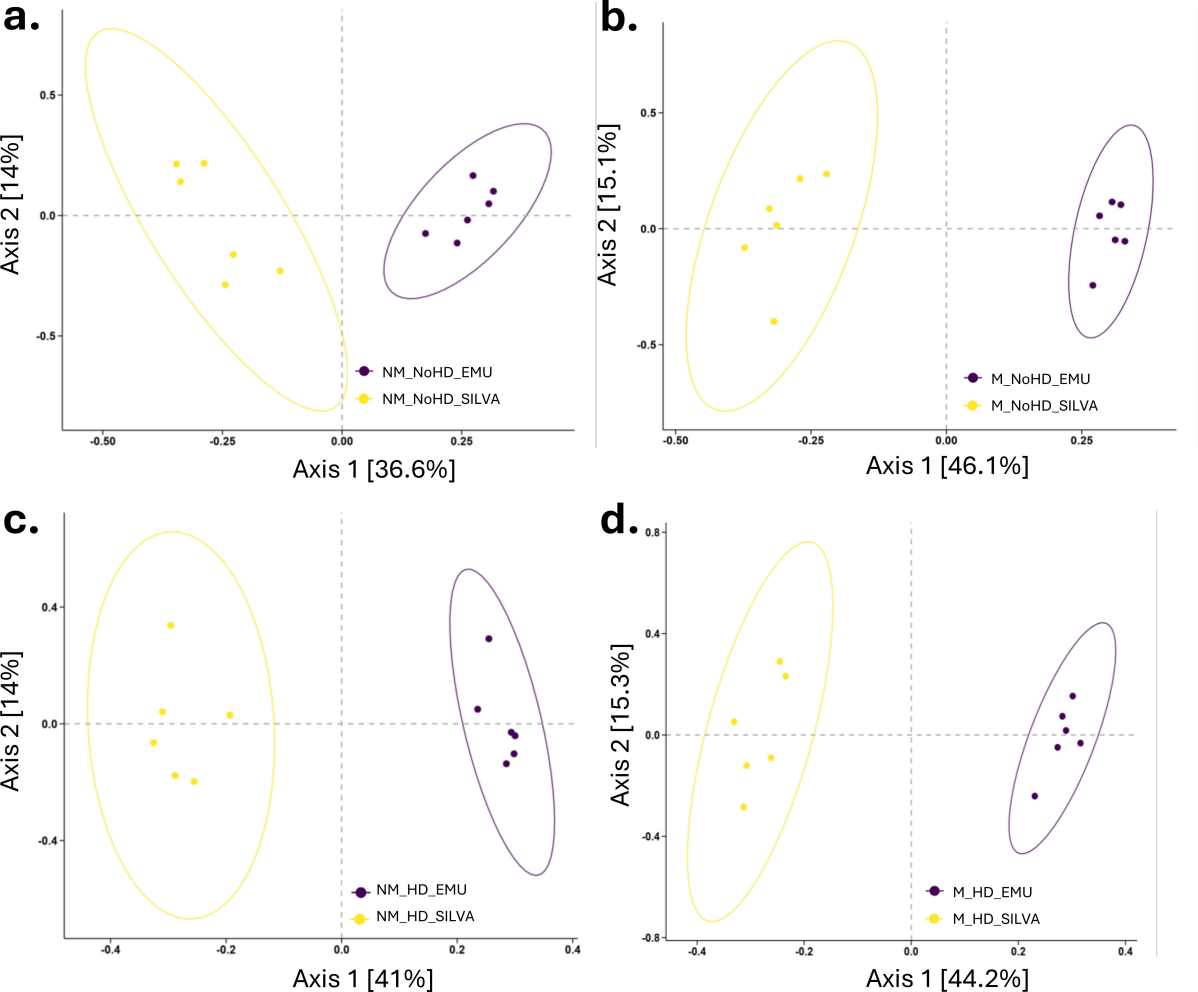
Beta diversity is significantly different between the Emu and SILVA reference databases**.** The principal coordinate analysis (PCoA) plots are based on the Unweighted UniFrac distance-matrix at the species level. Comparison of reference databases (Emu vs. SILVA) for (**a**) no medium samples without host depletion, (**b**) medium samples without host depletion, (**c**) no medium samples with host depletion, and (**d**) medium samples with host depletion. All comparisons here were on species taxonomic level. Significant differences in beta diversity (Bray-Curtis dissimilarity index and Unweighted Unifrac) were found at all taxonomic levels using a permutational ANOVA (PERMANOVA) test with 9,999 permutations (*P* < 0.05).

However, beta diversity analysis using Bray-Curtis dissimilarity and Unweighted UniFrac showed significant community differentiation between the Emu and SILVA reference databases on all taxonomic levels (PERMANOVA test, 9,999 permutations, all *P*-values < 0.05) (Fig. 5).

In addition, SILVA, with its larger and broader data set, identified more taxa across all taxonomic levels, detecting 10 phyla (compared to eight in Emu), 79 families (versus 49 in Emu), 182 genera (versus 125 in Emu), and 262 species (versus 211 in Emu).

However, the broader taxonomic coverage of SILVA came at the cost of lower species-level resolution. Specifically, 160 out of 262 taxa (61%) could not be classified beyond the genus level (i.e., reported as *Genus* sp.) when using SILVA compared to 0 out of 211 taxa (0%) with Emu.

**Fig 6:**
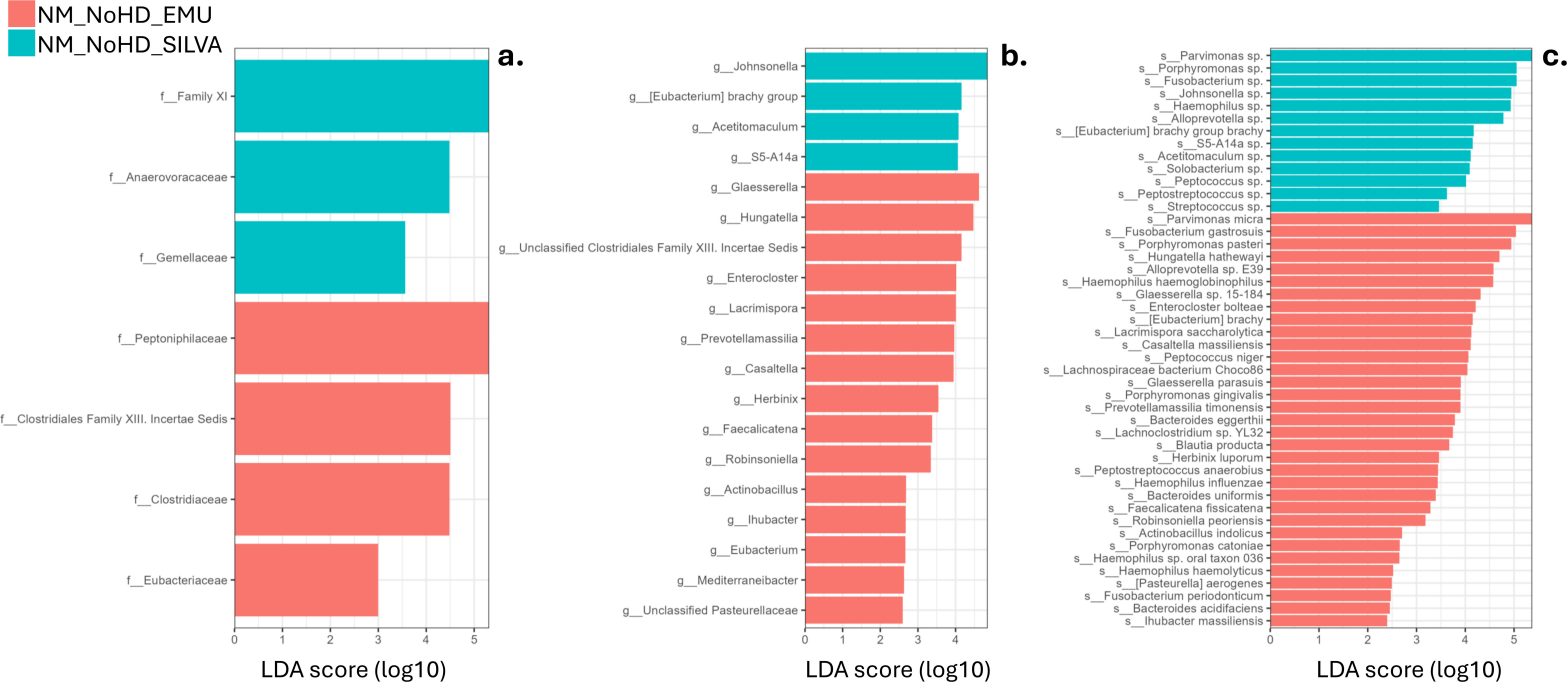
Differentially abundant bacterial taxa identified between the Emu and SILVA database using LEfSe. The LEfSe analysis was conducted to compare Emu and SILVA within each group individually. This figure presents the results for the no medium group without host depletion, highlighting significant taxa with an LDA log score > 2.0 at the family (**a**), genus (**b**), and species (**c**) levels.

Furthermore, LEfSe analysis showed multiple taxa that were significantly different between the two reference databases (LDA log score > 2) (Fig. 6).

In the other group comparisons, LEfSe analysis yielded similar results between the Emu and SILVA databases. Notably, *Lachnoclostridium* was significantly more abundant in host-depleted samples without medium when analyzed with Emu compared to SILVA, a difference not observed in other sample groups.

### *Porphyromonas*, *Parvimonas*, and *Fusobacterium* are most frequently found in canine vaginal samples

**Fig 7:**
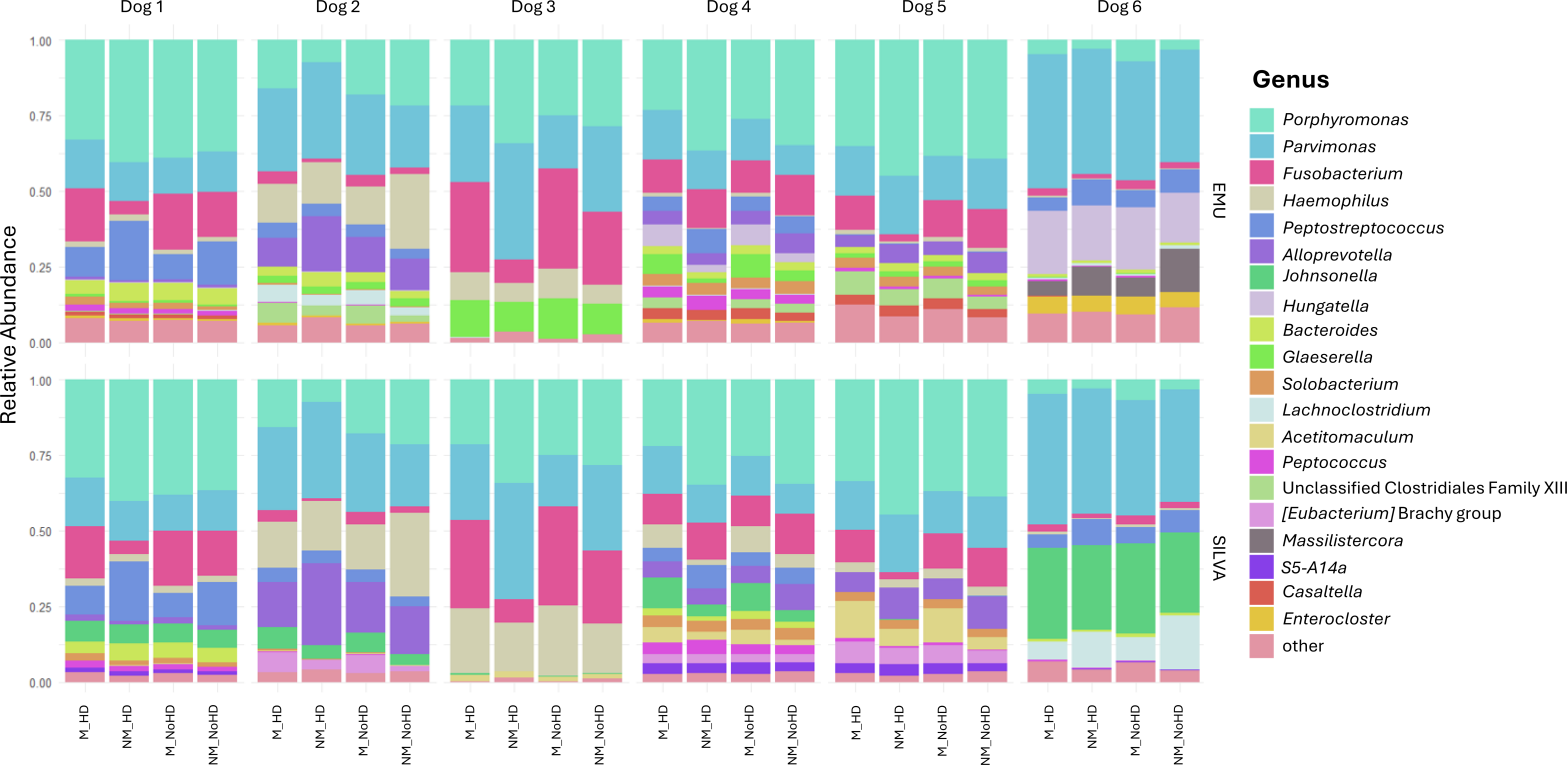
*Porphyromonas*, *Parvimonas*, and *Fusobacterium* are most frequently found in vaginal samples of clinically healthy bitches. Relative abundance of the 20 most abundant bacterial genera detected via 16S rRNA gene amplicon sequencing from vaginal swabs of healthy bitches in estrus, close to ovulation. Each dog was sampled twice: one swab was preserved in 2 mL of eNAT medium (Copan, Brescia, Italy) and the other in a sterile, PCR-clean 1.5 mL microcentrifuge tube (Eppendorf, Hamburg, Germany). Each sample was split into two aliquots, with one undergoing DNA extraction with host DNA depletion and the other processed without host DNA depletion. The plot is organized by individual dog, with the four sampling/processing conditions displayed side by side for comparison. The upper row displays results from sequences compared against the Emu database, while the lower row presents results from sequences compared against the SILVA database (M = medium, NM = no medium, HD = host depletion, NoHD = no host depletion).

The four most abundant phyla when using the Emu database were *Firmicutes* (47%), *Bacteroidetes* (32.9%), *Fusobacteria* (11.5%), and *Proteobacteria* (8.5%) and those when using the SILVA database were *Firmicutes* (47.5%), *Bacteroidota* (32.5%), *Fusobacteriota* (11.3%), and *Proteobacteria* (8.5%). The two most abundant phyla in the two negative control samples were *Firmicutes* (74%) and *Proteobacteria* (20%) according to both databases. The three most abundant genera from the vaginal swabs when using the Emu database were *Porphyromonas* (25.8%), *Parvimonas* (23.1%), and *Fusobacterium* (10.7%), with the remaining genera each constituting less than 5% (Fig. 7). When using the SILVA database, these numbers were *Porphyromonas* (25.3%), *Parvimonas* (22.7%), and *Fusobacterium* (10.5%), with three more genera that reached abundances above 5%: *Haemophilus* (8.3%; Emu 4.6%), *Johnsonella* (7.9%; Emu 0%), and *Alloprevotella* (5.8%; Emu 3.9%).

The five most abundant species across all samples were *Parvimonas micra* (23%), *Fusobacterium gastrosuis* (9.8%), *Porphyromonas pasteri* (9.8%), *Porphyromonas gingivicanis* (7.3%), and *Porphyromonas cangingivalis* (7%) when compared to the Emu database. In contrast, classification against the SILVA database resulted primarily in a genus-level identification, with the designation “sp.” indicating no specific species information: *Parvimonas* sp. (22.8%), *Porphyromonas* sp. (10.7%), *Fusobacterium* sp*.* (9.8%), *Haemophilus* sp. (8.2%), and *Johnsonella* sp. (7.9%). However, some *Porphyromonas* sequences were identified to the species level, specifically as *Porphyromonas cangingivalis* (6.8%), *Porphyromonas canis* (3.7%), and *Porphyromonas gingivicanis* (3.4%).

## DISCUSSION

This study highlighted four key findings: (i) the canine cranial vagina is a low-biomass environment; (ii) storage methods (medium versus no medium) had limited impact on DNA yield and microbial composition; (iii) host DNA depletion did not significantly alter microbial profiles nor selectively deplete gram-negative bacteria; and (iv) database choice (Emu versus SILVA) significantly influenced beta diversity and the number of detected taxa.

### Microbial composition differences across studies: sampling, storage, and methodology factors

Our study revealed a high relative abundance of *Porphyromonas* (25.3–25.8%), *Parvimonas* (22.7–23.1%), and *Fusobacterium* (10.5–10.7%) in canine vaginal samples. In contrast, Lyman et al. (4) reported *Hydrotalea*, *Ralstonia*, and *Mycoplasma* as dominant taxa, collectively accounting for 59.4% of the vaginal microbiota, though they also identified *Fusobacterium*, *Porphyromonas*, and *Parvimonas* at lower abundances (5–10%) (4). Notably, *Hydrotalea* and *Ralstonia* were absent from our samples. Rota et al. (6) found *Mycoplasma* (13.9%), an unidentified genus of the *Pasteurellaceae* family (7.84%), and *Salmonella* (7.6%) as the most abundant genera in anestrous bitches (6). In our study, *Mycoplasma* represented only 0.023% of the overall abundance, and *Salmonella* was not detected. Similarly, Hu et al. (5) identified *Fusobacterium* (~20%) as the most abundant genus in dioestrous bitches, followed by *Lactobacillus* and *Streptobacillus* (~10% each) (5). In comparison, *Streptobacillus* and *Lactobacillus* accounted for 0.3 and 0.003%, respectively, in our samples.

The marked differences in microbial profiles likely stem from factors, such as sampling technique, storage conditions, and DNA extraction protocols. For example, Rota et al. (6) stored samples at 4°C, while our samples were ultra-frozen at −80°C, which may affect bacteria like *Mycoplasma*, which are more susceptible to degradation (24). Variations in PCR methods, sequencing approaches, and reference databases further influence the results. Moreover, our study included only healthy, client-owned bitches in estrus, whereas other studies included dogs in different stages of the cycle or from shelter populations, which may differ in terms of overall health and microbial exposure. Additional differences in breed, living environment, and geographical origin of the dogs may also contribute to the discrepancies observed across studies.

### Contamination can be controlled using a double-guarded swab system

The canine cranial vagina is a low-biomass environment, with DNA yields ranging from 0.0352 to 4.49 ng/µL, while negative controls yielded 0.0057 to 0.0334 ng/µL. These findings highlight the importance of contamination control, particularly in low-biomass microbiome research. To minimize contamination, we employed a double-guarded swab system, ensuring sterile sampling of the cranial vagina and avoiding bacteria from the vulva, vestibulum, or caudal vagina.

Previous studies, such as that by Lyman et al. (4), reported higher DNA yields, which could be attributed to different sampling techniques, including the use of an otoscope cone as a covering (4). This method may have resulted in more contamination from the caudal vagina, potentially influencing the observed DNA concentrations. Other studies have not reported DNA yields or storage conditions (5, 6), making comparisons impossible.

Unlike the human vaginal microbiome, which is *Lactobacillus*-dominated and less diverse (25), the canine vaginal microbiome includes bacteria common to other body niches, such as the gastrointestinal tract and the skin. This higher diversity amplifies the potential impact of contamination during sampling, underscoring the need for stringent contamination control methods (8). In humans, vaginal microbiome samples are often self-collected at home without protective coverings on the swabs to enhance practicality and increase participation rates in studies (26, 27). However, such methods may be less applicable to the canine microbiome, given its greater diversity and higher susceptibility to contamination. Additionally, the anatomical structure of the canine vagina, which is characterized by a steep upslope, followed by a near-horizontal orientation, makes self-sampling by owners impractical and potentially hazardous, as improper technique could lead to swab breakage or injury to the dog.

Therefore, the use of a double-guarded swab system is recommended to ensure precise sampling of the cranial vagina with as little contamination as possible in canine microbiome research.

### Storage and processing conditions have limited impact on microbial profiles

Microcentrifuge tubes have been used for sample storage in the previous microbiome studies conducted by our research group (7), as well as in a study on the canine vaginal microbiome (4). They are cost-effective and widely available and allow for compact storage. However, placing a swab in a microcentrifuge tube requires cutting the swab to fit, introducing a potential source of contamination. Additionally, storing the swab dry necessitates the addition of a medium during sample processing, which introduces another potential contamination risk. Hence, we compared dry storage with storage in a specialized medium designed to stabilize DNA for microbiome studies.

In the present study, no significant differences were observed between medium and no medium storage conditions in terms of the DNA yield, number of reads, or comparisons of alpha- and beta-diversities. Ahannach et al. (2021) found that the eNAT medium preserves microbial composition consistently, even when samples from the same individual were stored under different conditions, demonstrating its ability to maintain sample integrity in varying circumstances (12). In the present study, samples from the same bitch were stored at −80°C for an identical time period; thus, the effects of storage time and temperature were not evaluated. While the impact of storage conditions on microbiome composition has yet to be studied for microcentrifuge tubes, previous research has demonstrated that the eNAT medium maintains microbial composition stability for at least 3 weeks at 4°C and an additional 3 days at room temperature (12). Additionally, the eNAT system allows for sterile handling by breaking the swab at a designated breakpoint directly into the sterile medium, ensuring the swab remains in a controlled, wet environment. Therefore, we consider the eNAT medium to be the preferred storage method in studies on the canine vaginal microbiome.

### Host depletion did not selectively reduce gram-negative bacteria

Ahannach et al. (2021) identified a bias in host-depleted samples of the female vaginal microbiome, where the relative abundance of gram-negative bacteria was significantly reduced (12). Their study employed multiple host depletion methods, including the QIAamp DNA Microbiome Kit, the HostZERO Microbial DNA Kit, and a protocol combining PMA treatment with the QIAamp PowerFecal Kit, while non-host-depleted samples were processed using only the PowerFecal Kit. In contrast, previous studies on the canine vaginal microbiome (4, 6) used the QIAamp DNA Mini Kit, which does not include a host depletion step. Thus, the absence of *Escherichia* sequences in these studies cannot be attributed to host DNA depletion. Moreover, no direct comparisons between host- and non-host-depleted samples have been performed in canine studies. In this study, we found no significant differences in alpha or beta diversity between host- (benzonase-treated) and non-host-depleted samples, nor did the LEfSe analysis identify any bacterial taxa as significantly different between the two extraction methods. This lack of distinction may be due to the milder nature of benzonase treatment compared to more aggressive host depletion methods, such as the HostZERO Kit.

*Escherichia coli* had a low relative abundance (0.12 [Emu]–0.19% [SILVA]) primarily detected in a single dog (dog 6). These findings suggest that *Escherichia* is not a dominant member of the vaginal microbiota of healthy bitches, and its frequent isolation by culture likely reflects its ease of growth rather than its ecological significance, highlighting the limitations of culture-based techniques in microbial community characterization.

Bacterial DNA extraction methods significantly impact the outcomes of microbiome research, influencing the apparent composition of microbial communities. In human microbiome research, especially through the Human Microbiome Project (HMP) (28), large cohort studies have demonstrated that sample collection (29, 30), DNA extraction methods (31, 32), library preparation, and sequencing (33, 34) can each substantially affect results. Standard operating procedures for fecal DNA extraction have been provided by the International Human Microbiome Standards (IHMS), noting that no single DNA extraction protocol outperforms across all criteria, such as repeatability, accuracy, and DNA yield and quality (35). This highlights the importance of tailoring extraction protocols to sample characteristics in individual studies.

In our study, we used the ZymoBIOMICS DNA Miniprep Kit, a widely used commercial kit in microbiome research designed for efficient bacterial DNA recovery. This kit employs both mechanical and chemical lyses to break open microbial cells, including those with tough cell walls. However, like all extraction methods, it introduces potential biases. Prior studies have noted that some extraction kits favor gram-negative bacteria, such as *Proteobacteria*, due to differences in lysis efficiency (36). Additionally, commercial kits, including the ZymoBIOMICS Kit, may introduce low-level contaminant DNA referred to as “kitome,” reinforcing the need for contamination controls in microbiome studies (37).

Given that we only used a single extraction kit and focused on comparing its performance with and without host DNA depletion, our findings do not provide insights into how different kits might influence microbial profiles. Future studies comparing multiple extraction methods would help determine whether specific bacterial taxa are underrepresented or overrepresented due to extraction biases. Standardizing DNA extraction within a study remains essential to ensuring comparability and minimizing methodological biases in microbial diversity and composition estimates.

### Primer choice/16S rRNA gene region and sequencing technology significantly impact taxonomic resolution

The choice of primers and the 16S rRNA gene region sequenced significantly impact microbial composition and resolution (38). The 16S rRNA gene includes conserved regions for primer binding and variable regions (V1 to V9) that differentiate bacterial taxa. Variations in primer binding can lead to biased amplification of certain bacteria. For example, a study on human gut samples showed that microbial profiles varied depending on the primer used, particularly at lower taxonomic levels (39). In vaginal microbiome research, certain regions like V1–V2 underestimated relevant bacteria, while V3–V4 provided a broader coverage of dominant genera, such as *Lactobacillus* and *Gardnerella* (40, 41). Additionally, sequencing platform differences, such as read length and error rate, can affect results. Previous canine vaginal microbiome studies employed V3–V4 in two cases (5, 6) and V4 (4) alone in another. However, none of these studies reported species-level classification, instead reporting only up to the genus level. This limitation likely arises from the short-read sequencing approaches used, which lack the resolution needed to distinguish closely related species.

In this study, Oxford Nanopore technology was utilized for full-length 16S rRNA sequencing, aiming to achieve more accurate species-level classification compared to previous studies. Full-length sequencing provides greater taxonomic resolution by incorporating all nine variable regions (V1–V9), reducing the risk of species misclassification seen in short-read methods (42). This is particularly important for closely related species, such as *Mycoplasma*, which may differ in their association with health or disease (43).

### The choice of database significantly influenced taxonomic resolution

The accurate identification of sequences through reference databases is critical in microbiome research, but the variability among databases introduces challenges for comparisons. To enhance uniformity in canine vaginal microbiome studies, we assessed two reference databases: SILVA containing 411,933 curated sequences and Emu with 49,301 sequences. SILVA, which is widely used in short-read sequencing, offers greater taxonomic resolution, identifying a broader range of taxa, especially at the genus level (16, 44, 45). In contrast, Emu, which is designed for species-level profiling with Oxford Nanopore full-length 16S rRNA gene sequencing, is a more specialized but smaller database (17).

When comparing these databases, we found similar classification outcomes at the phylum, family, and genus levels, with a consistent identification of dominant genera, such as *Parvimonas*, *Porphyromonas*, and *Fusobacterium*, across both databases. However, certain genera were uniquely identified by each: for example, *Johnsonella* (7.9% average abundance) was detected by SILVA but not Emu, while *Hungatella* (4% abundance) was identified by Emu but not SILVA. At the species level, differences between the databases reflect their design rather than a simple ranking of performance. Emu contains fewer sequences, but most belong to well-described species, increasing the likelihood of obtaining a species-level classification when a sequence matches its reference set. In contrast, SILVA includes a larger number of sequences per genus, including those from less-characterized or undescribed species, which results in a higher proportion of sequences being classified only at the genus level.

For example, sequences classified as *Parvimonas* sp. using SILVA were assigned specifically to *Parvimonas micra* when using the Emu database. Although *P. micra* is present in both databases, the SILVA-based classification did not resolve the read to the species level likely due to its broader, more conservative taxonomic annotation strategy. This case illustrates how the same underlying sequence can yield different results depending on the database’s taxonomic resolution and curation approach. These differences underscore the importance of selecting a database that aligns with the study’s objectives, whether prioritizing species-level resolution or broader taxonomic representation.

The size and content of each database significantly impacted the classification results. SILVA, with its larger and broader data set, identified more taxa across all taxonomic levels. Additionally, database nomenclature varied, with Emu using "Actinobacteria" and "Bacteroidetes," while SILVA referred to these as "Actinobacteriota" and "Bacteroidota," reflecting its adoption of the more recent nomenclature for bacterial phyla (46). These differences underscore the importance of carefully considering the database composition, nomenclature, and updates when comparing studies. Variations between databases can lead to discrepancies in taxonomic identification, highlighting the need for caution when drawing conclusions from studies that use different databases. Nevertheless, both databases consistently identified the most prevalent taxa within the vaginal microbiome, indicating that key bacterial groups can still be reliably detected despite differences in classification.

### Conclusion

Based on our findings, we propose a set of guidelines to enhance the reliability of microbiome research on the canine vaginal microbiome. Given the susceptibility of such studies to contamination and technical variability, a careful protocol design is essential. Samples from the cranial region of the vagina can be effectively collected using a double-guarded swab technique with sterile gloves to reduce contamination risk. Placing swabs in eNAT medium helps to stabilize bacterial DNA, while DNA extraction without host depletion provides consistent results. Sequencing with Oxford Nanopore technology offers the advantage of long-read sequencing for species-level resolution. The SILVA database provides a broad and reliable reference for taxonomic assignment due to its extensive data set, while tools like EMU can further refine species-level identification. The choice of database should be guided by the specific research question to ensure optimal accuracy and resolution. These practices are confirmed by our research and offer a flexible framework to minimize variability and improve reproducibility in future studies.

### Limitations of the study

While this study provides valuable insights, there are some aspects that warrant further exploration. The sample size comprising 26 samples from six dogs and the range of storage conditions tested were limited. Additionally, the impact of long-term storage and storage temperature was not assessed, and future studies should evaluate extended storage periods with multiple time points and storage temperatures. Our research focused primarily on bacterial communities, and while this was a key objective, future investigations could expand to include viruses and fungi. Lastly, although two DNA extraction protocols were tested, further research could explore a wider range of extraction techniques and kits to optimize outcomes.

An additional limitation relates to contamination removal in low-biomass samples. Due to the limited number of negative controls (*n* = 2), it was not possible to apply a statistically robust method, such as the decontam R package, which requires either multiple controls (for prevalence-based filtering) or a broader range of DNA concentrations (for frequency-based filtering). As an alternative, we subtracted the read counts of taxa detected in the negative controls from the corresponding taxa in the actual samples. To minimize the impact on data integrity, this approach was applied conservatively, and any taxa removed entirely from a sample were confirmed to represent less than 1% of total reads. While this pragmatic solution ensured minimal distortion of community profiles, future studies should include a greater number of negative controls and implement statistically validated contamination detection methods to more rigorously account for background contamination in low-biomass environments.

To ensure accuracy in microbial identification, validating this protocol with mock communities would be ideal. However, the primary goal of this study was to create a standardized protocol for research on the canine vaginal microbiome, enabling more reliable comparisons across studies.

## Data Availability

All data supporting the findings of this study are included in the paper. Raw sequencing reads generated during this study have been deposited in the NCBI Sequence Read Archive (SRA) under BioProject ID [PRJNA1270378]. Additional data and materials can be made available upon request from the corresponding author.
